# Observation of quantum depletion in a non-equilibrium exciton–polariton condensate

**DOI:** 10.1038/s41467-019-14243-6

**Published:** 2020-01-22

**Authors:** Maciej Pieczarka, Eliezer Estrecho, Maryam Boozarjmehr, Olivier Bleu, Mark Steger, Kenneth West, Loren N. Pfeiffer, David W. Snoke, Jesper Levinsen, Meera M. Parish, Andrew G. Truscott, Elena A. Ostrovskaya

**Affiliations:** 1 ARC Centre of Excellence in Future Low-Energy Electronics Technologies and Nonlinear Physics Centre, Research School of Physics, The Australian National University, Canberra, ACT 2601 Australia; 2 ARC Centre of Excellence in Future Low-Energy Electronics Technologies and School of Physics and Astronomy, Monash University, Melbourne, VIC 3800 Australia; 3 Department of Physics and Astronomy, University of Pittsburgh, Pittsburgh, PA 15260 USA; 4 Princeton Institute for the Science and Technology of Materials (PRISM), Princeton University, Princeton, NJ 08544 USA; 5 Laser Physics Centre, Research School of Physics, The Australian National University, Canberra, ACT 2601 Australia; 6 Present Address: National Renewable Energy Lab, Golden, CO 80401 USA

**Keywords:** Bose-Einstein condensates, Quantum fluids and solids

## Abstract

Superfluidity, first discovered in liquid ^4^He, is closely related to Bose–Einstein condensation (BEC) phenomenon. However, even at zero temperature, a fraction of the quantum liquid is excited out of the condensate into higher momentum states via interaction-induced fluctuations—the phenomenon of quantum depletion. Quantum depletion of atomic BECs in thermal equilibrium is well understood theoretically but is difficult to measure. This measurement is even more challenging in driven-dissipative exciton–polariton condensates, since their non-equilibrium nature is predicted to suppress quantum depletion. Here, we observe quantum depletion of a high-density exciton–polariton condensate by detecting the spectral branch of elementary excitations populated by this process. Analysis of this excitation branch shows that quantum depletion of exciton–polariton condensates can closely follow or strongly deviate from the equilibrium Bogoliubov theory, depending on the exciton fraction in an exciton polariton. Our results reveal beyond mean-field effects of exciton–polariton interactions and call for a deeper understanding of the relationship between equilibrium and non-equilibrium BECs.

## Abstract (web summary)

Many aspects of polariton condensate behaviour can be captured by mean-field theories but interactions introduce additional quantum effects. Here the authors observe quantum depletion in a driven-dissipative condensate and find that deviations from equilibrium predictions depend on the excitonic fraction.

## Introduction

The fundamental understanding of interacting cold bosonic gases was developed by N. Bogoliubov, whose theory predicted the consequences of interparticle interactions for the properties of Bose–Einstein Condensates (BECs)^1^. According to this theory, an interacting BEC^2^ is characterised by a modified phonon-like dispersion of elementary excitations at long wavelengths (or short wavevectors) that explains the superfluid properties of weakly interacting BECs. The crux of the theory is the non-perturbative transformation of Bogoliubov quasiparticles, where a condensate excitation at a given momentum is expressed as a superposition of counter-propagating single-particle states^3^. The quantum fluctuations of the interacting particles in the ground state are responsible for the non-zero occupation of these elementary excitations at zero temperature, leading to the so-called quantum depletion of the condensate population. Quantum depletion was observed in weakly interacting BECs^4–6^ of ultra-cold atoms and is the main reason for a low condensed fraction of strongly interacting superfluid ^4^He^7^. This effect is predicted to have pronounced experimental signatures, where the occupation of elementary excitation modes exhibits a distribution in momentum space *N*(*k*) scaling as *k*^−4^ for long wavevectors. This behaviour is challenging to observe in weakly interacting atomic BECs^5,6,8^, because the momentum distribution is not preserved in the time-of-flight measurements and is influenced by interactions during the expansion of the condensate^9^.

Exciton–polariton condensates, which are part-light part-matter bosonic condensates formed in a semiconductor microcavity, allow direct measurement of their momentum space distribution and excitations through the cavity photoluminescence signal. Each exciton polariton (or polariton) is a strongly coupled quantum well exciton and a cavity photon. Due to the finite lifetime of the confined photon state, the polariton eventually decays and a photon escapes the microcavity retaining the energy and momentum of the polariton^10^. The finite lifetime results in the inherent driven-dissipative nature of exciton–polariton condensates, where the system needs constant pumping to maintain the condensate population, hence the steady state is reached based on a balance between driving and dissipation. In addition, the finite lifetime often prevents full thermalisation of the condensate, which is typically manifested by a macroscopic occupation of several single-particle energy states rather than a single ground state. Furthermore, the constant energy flow in exciton–polariton condensates strongly influences the elementary excitation spectrum, which can possess a gapped mode or exhibits a flat dispersionless Goldstone mode at the low energy limit, gradually recovering the textbook Bogoliubov dispersion at longer wavevectors^11^. Nonetheless, exciton–polariton condensates preserve superfluid properties^12,13^, although their features are currently understood as a rigid state under continuous coherent driving^14^.

The striking feature of the Bogoliubov excitation spectrum is the appearance of two branches which are positive (normal branch—NB) and negative (ghost branch—GB) with respect to the condensate energy. While occupation of the NB occurs via the process of thermal excitation (thermal depletion), the GB is populated solely by the quantum depletion process, thus its appearance in the photoluminescence spectrum can serve as a direct probe of beyond mean-field effects (quantum fluctuations) in an exciton–polariton condensate^15^. Quite notably, the quantum effects of polariton–polariton interactions have been recently demonstrated, but at the single-particle level, through correlation experiments on strongly confined exciton polaritons^16–18^. There have been several attempts to measure the full excitation spectrum of exciton–polariton condensates including populating both excitation branches using resonant pump–probe schemes^19–21^ and intense incoherent driving^22,23^. However, none of these approaches created the condensates in a spontaneous, steady-state configuration. More importantly, the population of the GB was forced by scattering on defects^22^ or by an additional resonant laser beam^19^, hindering the direct observation of interaction-driven many-body quantum effects.

The linear Bogoliubov spectrum of a spontaneously created high-density exciton–polariton condensate has been observed, but only the positive energy NB photoluminescence was visible in the momentum space and its population was naturally dominated by thermal excitations of the condensate^24^, which scaled as *k*^−2^ in the low wavevector limit. Recently, the problem of reduced visibility of the GB was tackled theoretically^15,25^, showing that non-equilibrium effects can suppress the quantum depletion and its signatures in the photoluminescence spectrum of an exciton–polariton condensate.

In this work, we create a steady-state high-density condensate of long-lifetime exciton polaritons in an ultrahigh-quality GaAs-based microcavity, and observe the direct manifestation of quantum depletion in the condensate excitation spectra (see “Methods”). This is achieved by an optical excitation scheme, where a non-resonant pump laser beam with an annular spatial distribution^26,27^ creates a similarly shaped distribution of incoherent excitonic reservoir particles that provides both gain and a trapping potential for the exciton polaritons^28^. At a sufficiently high pump power, a single-mode condensate in the interaction-dominated Thomas–Fermi regime^27^ forms inside the trap away from the pump-induced potential barrier. The spatial separation of the pump and the condensate allows us to filter out the photoluminescence (see “Methods”) originating from regions with significant overlap with the reservoir and analyse the emission of the condensate and its excitations. In addition, the single-mode condensate ensures that we are dealing with a simple many-body macroscopically occupied ground state of the pump-induced trap. To test both the equilibrium and non-equilibrium features of the exciton–polariton condensate, we study the system at various values of detuning between the cavity photon and the exciton energies Δ = *E*_C_−*E*_X_. A wide range of detuning values is accessible via the cavity wedge (see “Methods”), and reflects either a more matter-like (excitonic) or a light-like (photonic) nature of the polaritons at positive or negative detunings, respectively. In what follows, we present full datasets for two representative values of detuning: positive (Δ = +1.8 meV) and negative (Δ = −3.7 meV). The Hopfield coefficients^10^ determining the exciton (matter) fraction of the polariton at these detunings are |*X*|^2^ ≈ 0.56 and |*X*|^2^ ≈ 0.39 at *k*_∥_ = 0, respectively. The excitonic or photonic nature of exciton polaritons affects their ability to achieve thermal equilibrium^29,30^.

## Results

### Condensation in an optically induced trap

**Fig. 1:**
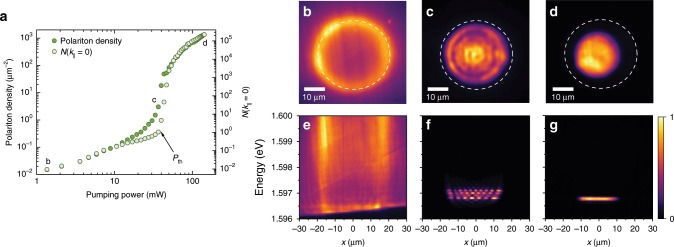
High-density single-mode condensation. **a** Pump power dependence of the total mean polariton density (filled circles) and the occupation number, *N*(*k*_||_), of the *k*_||_ = 0 state (empty circles), both measured inside the trap. Ground state condensation threshold *P*_th_ is indicated with an arrow. **b**–**d** Energy integrated, real space images of exciton–polariton luminescence at the three density regimes marked in panel (**a**) shown together with (**e**–**g**) the corresponding real space spectra taken in the middle of each image. **b**, **e** Images far below condensation threshold, *n* < 0.02 μm^−2^. **c**, **f** Multimode condensation in the intermediate regime, *n* = 18 μm^−2^. **d**, **g** High-density, interaction-dominated single-mode regime, *n* = 1340 μm^−2^. Dashed circle in **b**–**d** represents the shape of the ring excitation. The colour scale in **b**–**g** is linear and represents photon counts per second (cts s^−1^) normalised to 1. Data are presented for the photonic detuning, Δ = −3.7 meV (see Supplementary Note 1 for the excitonic detuning data).

In our experiment, we utilise a continuous wave (CW) laser and create a steady-state polariton condensate. Nevertheless, condensation features are very similar to those observed in the pulsed excitation regime^27^. The typical condensate spectra are presented in Fig. 1 together with the dependence of the polariton density and the ground state occupancy on pump power (Fig. 1a). Below the condensation threshold, laser-induced high-energy excitons relax and form exciton polaritons with large wavevectors and energies, which create a local potential barrier and dominate the photoluminescence under these low-power excitation conditions, Fig. 1b, e. With increasing pump power, exciton polaritons increase in density until they condense into a fragmented condensate with the macroscopic occupation of several modes of the trap, see Fig. 1c, f. This fragmentation is due to the weak polariton–polariton interaction and inefficient phonon-mediated relaxation processes at intermediate densities^31,32^. Multimode condensation manifests itself in a smooth non-linear rise in polariton density at pumping powers slightly before single-mode condensation sets in, see Fig. 1a. After reaching the threshold at large polariton densities, stimulated scattering and efficient energy relaxation drive the condensation towards a single-mode ground state condensate with a spatially homogeneous density distribution and well-defined energy, as seen in Fig. 1d, g and Supplementary Notes 1 and 2. In this interaction-dominated Thomas–Fermi regime, we can reliably measure the spectrum of condensate excitations, and perform a direct comparison with the Bogoliubov theory.

### Condensate excitations and polariton–polariton interaction

**Fig. 2:**
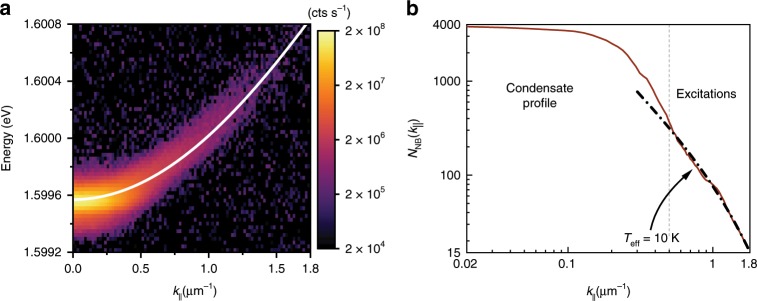
Momentum space photoluminescence of a polariton condensate. **a** Momentum space spectrum of the exciton–polariton condensate at the excitonic detuning (Δ = + 1.8 meV), where excitations can be detected together with the strong signal from the *k*_||_ = 0 condensate for *n* = 70 μm^−2^. Solid line is the single-particle (non-interacting) polariton dispersion. The colour scale is logarithmic. **b** Extracted momentum space occupation of the condensate and the thermal excitations in the normal branch in log–log scale. Dashed-dotted line indicates the profile of thermal excitations fit with the effective temperature *T*_eff_ ≈ 10 K.

In order to investigate the excitations of the steady-state exciton–polariton condensate, we measure the energy-resolved far-field emission of the condensate, which corresponds to the momentum- and energy-resolved distribution of exciton polaritons. Figure 2a presents the excitation spectrum of a single-mode condensate at intermediate densities, where the excitation spectrum is indistinguishable from the single-particle dispersion, i.e. the condensate interaction energy is too small to modify considerably the Bogoliubov spectrum within the linewidth of the photoluminescence signal. The result of the momentum space integration is presented in Fig. 2b in double-logarithmic scale. One can distinguish the condensate profile (the strongest signal located near *k*_||_ = 0, where *k*_||_ is the polariton momentum in the plane of the quantum well) from the thermal excitations, which are visible at longer wavevectors and are fitted with a thermal distribution, Fig. 2b (see “Methods”). The condensate at *k*_||_ = 0 has a finite width in momentum space, which is a consequence of the spatial confinement in an optical trap^24,28^.

**Fig. 3:**
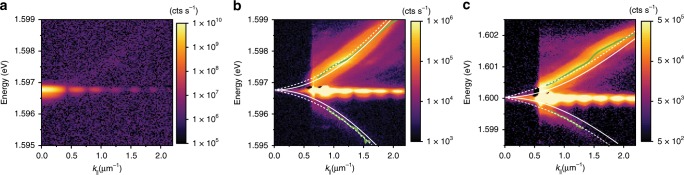
Measurement of the renormalized excitation spectrum. **a** Full photoluminescence of the high-density exciton–polariton condensate at the photonic detuning (Δ = −3.7 meV) and *n* ≈ 1340 μm^−2^. **b** Excitation spectrum of the polariton condensate in **a** measured with a momentum edge filter covering the signal up to *k*_||_ ≈ 0.55 μm^−1^. **c** Excitation spectrum measured at the excitonic detuning (Δ = + 1.8 meV) and *n* ≈ 1848 μm^−2^. Colour scales are logarithmic and images (**b**, **c**) are saturated. Green solid lines indicate extracted spectral positions of the branches. Solid white lines represent single-particle dispersions and dashed lines correspond to the renormalized Bogoliubov dispersions fitted to the data.

The momentum spectra differ significantly in the high-density regime around *n* ~ 10^3^ μm^−2^, where an exceptionally strong signal from the ground state exceeds all other contributions in the spectrum within the dynamical range of the CCD camera, see Fig. 3a. The condensate emission also reveals a characteristic Airy pattern due to diffraction of the condensate photoluminescence on a circular aperture we use as a real space filter to block the emission from the trap barrier (see “Methods” and Supplementary Note 3). To reveal the much weaker signal of the excitation branches, we impose an edge filter in momentum space, blocking the strongest contribution to the signal up to about 0.55 μm^−1^, Fig. 3b, c. Remarkably, the signal from the two branches becomes clear and distinguishable, despite the strong contribution of the diffracted condensate photoluminescence. The appearance of the GB signal is evidence of quantum depletion of the exciton–polariton condensate, being a consequence of quantum fluctuations in the steady state.

**Fig. 4:**
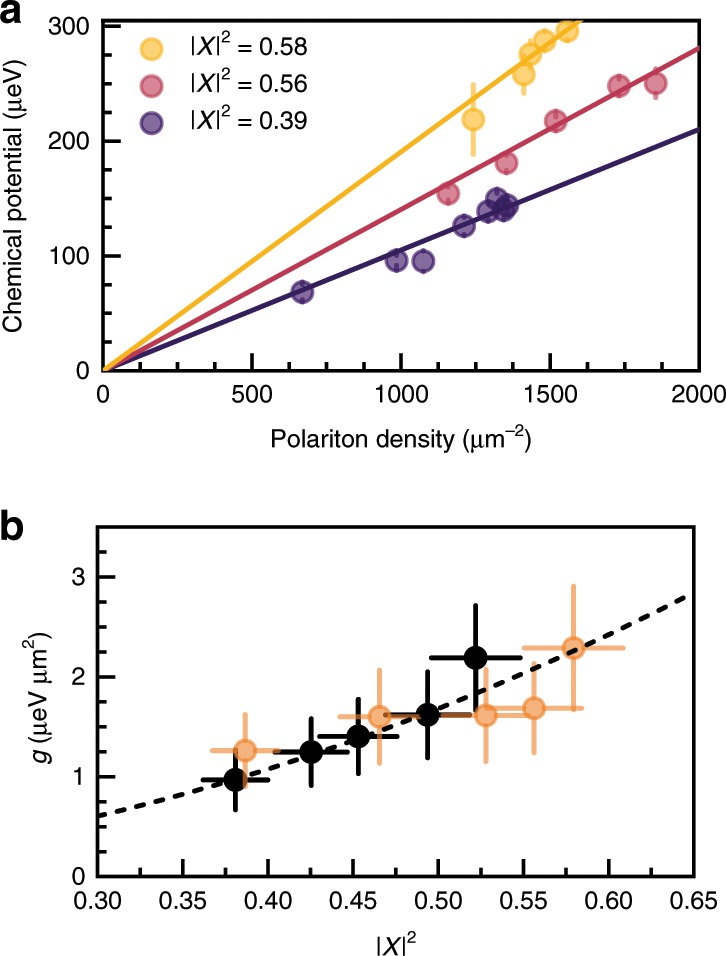
Polariton–polariton interaction. **a** Condensate energies (chemical potential μ = *gn*) extracted from Bogoliubov dispersion fitting for three different values of detuning (other detuning values are not presented for clarity of the plot). Error bars express the goodness of fit of the excitation spectrum. **b** Summary of extracted interaction strengths per single quantum well as a function of the excitonic Hopfield coefficient (exciton fraction). Orange dots represent data collected from fitting the GB dispersion, as described in the text. The data are in excellent agreement with values obtained from an independent experiment in a pulsed excitation regime^27^ (black dots). Fit to the data using a quadratic function of |*X*|^2^ is shown with a dashed line. Error bars represent the estimated inaccuracy of the detuning and the error originating from inhomogeneities of the condensate profile^27^.

Fitting the excitation branches dispersions, see Fig. 3b, c, with Eq. (2) (see “Methods” and Supplementary Notes 4 and 5) allows us to extract the interaction energy (chemical potential) μ of the condensate as a function of the condensate density, which follows closely the expected linear dependence μ = *gn*, where *g* is the polariton–polariton interaction strength and *n* is the measured polariton density, see Fig. 4a. This fitting procedure can be applied to the experimental data at various detunings, where the GB signal and the dispersion change of the excitation branches are observable. The extracted values of the polariton–polariton interaction strengths, normalised to the number of quantum wells, is presented in Fig. 4b, together with the values obtained under pulsed excitation and at larger densities (*n* ~ 10^4^ μm^−2^) in the Thomas–Fermi regime^27^. One observes an excellent agreement for all experimental values showing the theoretically predicted quadratic dependence on the exciton fraction: *g* ∝ |*X*|^4^*g*_X_, where *g*_X_ is the exciton–exciton interaction constant^10,27^, which in our case is *g*_X_ = 13.5 ± 0.6 μeVμm^2^. We note that, in contrast to ref. ^27^, the direct measurement of the interaction constant from the blueshift of the Thomas–Fermi condensate energy in the CW regime is not possible because of the incomplete depletion of the reservoir, which effectively lifts the zero-point energy of the optically induced potential (see Supplementary Note 5). The measurement of the interaction strength presented here is therefore methodologically different and independent from that in ref. ^27^.

### Occupation of the excitation branches in momentum space

**Fig. 5:**
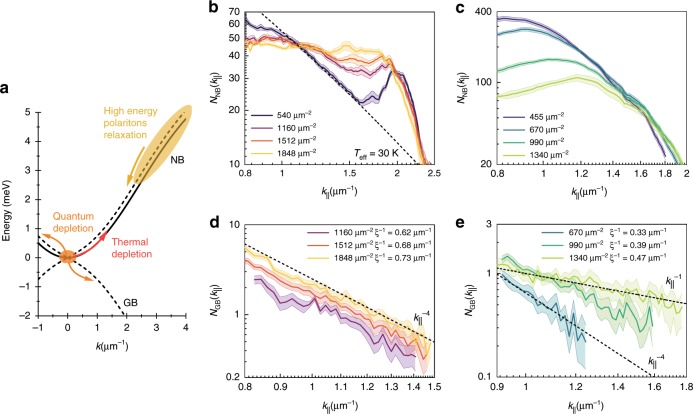
Depletion mechanisms—analysis of the extracted momentum space distributions. **a** Schematics showing the renormalised dispersions of the condensate excitations and possible occupation mechanisms due to quantum depletion, thermal depletion and relaxation of high-energy polaritons. Normal branch occupation for high-density polariton condensates at the (**b**) excitonic (Δ = + 1.8 meV) and (**c**) photonic (Δ = −3.7 meV) detunings. Dashed line in **b** is a guide to the eye reference of a thermal distribution of excitations at an effective temperature *T*_eff_ ≈ 30 K. High-*k*_||_ polaritons are more pronounced at higher densities for the photonic detuning. Ghost branch occupancies for (**d**) excitonic and (**e**) photonic detunings. For more excitonic polaritons, the GB occupation behaves according to the equilibrium theory, following the asymptotic power-law decay at larger momenta. At the photonic detuning, the GB occupation shows a deviation from the Bogoliubov theory. Similar behaviour is observed at other values of excitonic or photonic detuning, as shown in Supplementary Note 9. Plots (**b**–**e**) are in log–log scale and the calculated crossover wavevectors *k*_*ξ*_ = *ξ*^−1^ are indicated in the legends of **d** and **e**. Shaded zones in **b**–**e** represent error bars of the occupation numbers extraction taking into account fitting errors of the spectra at a given *k*_||_.

The essential information on the mechanisms populating the elementary excitation branches, summarised in Fig. 5, is contained in the characteristic momentum occupation distributions *N*(*k*_||_). The employed filtering technique allows us to measure these distributions in the long wavevector range, i.e. *k*_||_*ξ* > 1, where $\xi = \frac{\hbar }{{\sqrt {2mgn} }}$ is the healing length and *m* is the polariton effective mass (see Supplementary Note 6). In this wavevector range, the NB occupation is characterised by non-equilibrium features at both probed values of the exciton–photon detuning, see Fig. 5b, c. At densities *n* < 10^3^ μm^−2^, *N*_NB_ displays thermal-like distributions at higher momenta for the excitonic detuning. However, at the largest probed densities, thermal excitations are reduced, leading to a population of excited trap states for both photonic and excitonic polaritons, Fig. 5b, c. This is caused by the interplay of the different mechanisms populating the NB, as schematically depicted in Fig. 5a. In addition to thermal and quantum depletion of the condensate, the inefficient energy relaxation of polaritons naturally leads to an occupation of high-*k*_||_ states (see Supplementary Note 1). These high-energy polaritons originate from the potential barrier region (the reservoir) and are more prevalent for more photonic polaritons where the interactions and thermalisation are much weaker^10,29,30^. The peak around *k*_||_ = 2 μm^−1^ observed at the excitonic detuning in Fig. 5b originates from high-energy states on top of the pump region, which spread all over the area of the trap, and are similar to a high-energy state observed in a single spot excitation of a high-quality sample^33^. In addition, polariton buildup and accumulation at high-energy states may be linked to the polariton-to-reservoir upconversion mechanism, which has been observed at strong coherent pumping fields in multiple independent experiments and is a source of the incoherent reservoir generation within the trap^17,18,34–36^.

In contrast to the NB, the GB is populated solely by quantum fluctuations of the high-density polariton condensate. Therefore, one expects to observe clear signatures in the momentum space distribution as predicted by the Bogoliubov theory. Indeed, the occupation distribution shows a *k*^−4^ decay for the excitonic detuning, see Fig. 5d and Supplementary Notes 9 and 10 (henceforth *k* denotes the in-plane momentum *k*_||_).

This is in agreement with the Bogoliubov theory prediction of the asymptotic behaviour at large wavevectors and suggests the equilibrium-like character of the condensate quantum depletion despite the deviations caused by non-equilibrium effects in the NB distributions. In addition, we observe a predicted quadratic increase of the GB occupation as a function of the condensate density (see “Methods” and Supplementary Note 11). The most remarkable behaviour of the GB occupation distributions is observed at the photonic detuning, as seen in Fig. 5e and Supplementary Note 9. Here, in spite of the rising population density (and pump power), we observe a gradual transition from the *k*^−4^ distribution to a plateau approaching *k*^−1^. The deviations from the equilibrium distributions have been predicted by non-equilibrium theories^37–39^, indicating that at photonic detunings polariton condensate fluctuations are mediated mostly by reservoir–condensate interactions, and polariton–polariton interactions do not play a major role. This assertion is supported by the fact that the polariton–polariton interaction is weaker for more photonic polaritons (Fig. 4b), and that the reservoir density, *n*_R_, extracted from our measurements grows with pump power, with the condensate fraction, defined as *ρ* = *n*/(*n* + *n*_R_), reaching maximum values of *ρ* ≈ 0.5 (see Supplementary Note 5). Furthermore, as discussed in the Supplementary Note 12, condensates at photonic detunings might be subject to large reservoir-driven fluctuations, which could lead to departure from the Bogoliubov prediction for the GB occupation^39^.

It is important to note that the unusual momentum distributions *N*_GB_(*k*) observed at photonic detunings do not originate from the non-parabolic dispersion of polaritons. Taking into account the full polariton dispersion rather than the parabolic effective mass approximation, leads to a deviation from the *k*^−4^ behaviour of the population numbers, which is small within the experimentally accessed range of momenta (see “Methods” and Supplementary Note 13).

## Discussion

To summarise, our experiment represents a direct probe of quantum fluctuations and interparticle interactions in a many-body driven-dissipative BEC. In our exciton–polariton system, we observe a crossover from the near-equilibrium regime of quantum fluctuations at the excitonic detunings to a fully non-equilibrium regime at the photonic detunings, where reservoir fluctuations might play a critical role. Our experimental findings are beyond the current theoretical understanding of non-equilibrium condensates. Thus, they call for further development of a theory describing the quantum depletion of BECs in the crossover from thermal equilibrium to far-beyond equilibrium conditions, as well as detailed mechanisms responsible for population of both normal and ghost branches of Bogoliubov excitations in the non-equilibrium regime. Furthermore, in the regime where the equilibrium Bogoliubov theory of quantum depletion does apply, our experiment paves the way for the measurement of the Tanʼs contact^5,40^ for exciton–polariton condensates (see “Methods”). This, in turn, will allow us to test whether exciton–polariton condensates exhibit universal thermodynamical properties of a system with contact interactions^41^.

## Methods

### Experiment

The sample used in the experiment is a high-quality factor GaAs-based microcavity characterised by a long cavity photon lifetime exceeding 100 ps^42^. The 3*λ*/2 cavity consists of distributed Bragg reflectors with 32 (top) and 40 (bottom) pairs of alternating Al_0.2_Ga_0.8_As/AlAs layers and an active region of 12 GaAs/AlAs quantum wells of 7 nm nominal thickness positioned in three groups at the maxima of the confined photon field. The normal mode anti-crossing (Rabi splitting) is measured to be about *ћ*Ω = 15.9 ± 0.1 meV^43^, the exciton resonance energy is *E*_X_ = 1.6062 eV, and the cavity photon effective mass is about 3.6 × 10^−5^*m*_0_, where *m*_0_ is the free electron mass^27,44^. In all experiments the microcavity is kept in a continuous flow helium cryostat, ensuring the sample temperature of 7–8 K.

The non-resonant excitation in the experiment was provided by a single-mode CW Ti:Sapphire laser tuned to a reflectivity minimum of the microcavity (around 719 nm) for efficient photon absorption in the quantum wells. To minimise the thermal heating of the sample, the laser pump was chopped with an acoustic optical modulator at 10 kHz and 5% duty cycle. The ring-shaped excitation profile is created by utilising an axicon lens in a confocal configuration between two imaging lenses that produces a hollow beam^45^ when re-imaged onto the sample surface via a microscope objective of NA = 0.5. The same objective collects the photoluminescence from the sample. The imaging setup is composed of four lenses in confocal configuration for measurements of near and far-field emission image planes^26^. The image filtering is performed in the intermediate conjugate planes with an optical iris in real space and a movable razor-blade edge in momentum space. The filtered signal is then imaged onto the monochromator slit and dispersed by a grating, allowing measurement of spatial and momentum spectra of exciton–polariton photoluminescence. The signal is recorded by a high-efficiency EMCCD camera.

### Extraction of the occupation numbers in momentum space

Calculation of the momentum distribution is obtained based on the integration of the signal and taking into account the local density of states in momentum space^26^. The collection efficiency of the experimental setup was calibrated with a reference laser tuned to the emission wavelength of polaritons and is expressed as *η*. The mean number of polaritons recorded within a single pixel row on a CCD camera representing a wavevector *k*_*i*_ is calculated from the photon count rate $\frac{{dN_{{\mathrm{ph}}}(k_i)}}{{dt}}$:

$$N_{{\mathrm{pol}}}\left( {k_i} \right) = \eta \frac{{dN_{{\mathrm{ph}}}(k_i)}}{{dt}}\tau _{{\mathrm{LP}}}\left( {k_i} \right),$$

where *τ*_LP_ is the polariton lifetime calculated based on the Hopfield coefficients and cavity photon lifetime^10,26,46^. The occupation number of polaritons at a given *k*_*i*_ state is calculated taking into account the number of states subtended by a pixel at *k*_*i*_ position in cylindrical coordinates $N_{{\mathrm{st}}}\left( {k_i} \right) = k_i{\mathrm{\Delta }}k_i{\mathrm{\Delta }}\varphi _i \cdot \left( {\frac{{4{\uppi}^2}}{A}} \right)^{ - 1}:$

$$N\left( {k_i} \right) = \frac{{N_{{\mathrm{pol}}}\left( {k_i} \right)}}{{N_{{\mathrm{st}}}(k_i)}} = \frac{{4{\uppi}^2\eta }}{{2k_i{\mathrm{\Delta }}\varphi _i{\mathrm{\Delta }}k_iDA}}\frac{{dN_{{\mathrm{ph}}}\left( {k_i} \right)}}{{dt}}\tau _{{\mathrm{LP}}}\left( {k_i} \right).$$

The formula takes into account the volume of a single state in momentum space, spin degeneracy of 2, and the momentum space volume subtended by a single pixel. The duty cycle of the acousto-optic modulator is denoted by *D*, and the real space filter area is denoted by *A*. A detailed derivation of the integration formulas is given elsewhere^26^.

### Analysis of the Bogoliubov excitation branches

One can diagonalise a simplified Hamiltonian of a BEC of weakly interacting bosons using quasiparticle operators at a given wavevector^47^ *k*: $\hat b_k = u_k\hat a_k + v_{ - k}\hat a_{ - k}^\dagger$, which is a linear combination of creation and annihilation single-particle operators for a boson at given wavevector, $\hat a_k^\dagger,\, \hat{a}_{-k}$,  and the amplitudes are expressed as:

1 $$u_k,v_{ - k} = \pm \frac{1}{{\sqrt {2{\it{\epsilon }}\left( k \right)} }}\sqrt {E(k) + gn \pm {\it{\epsilon }}\left( k \right)} ,$$

where ${\it{\epsilon }}(k)$is the Bogoliubov dispersion in equilibrium

2 $${\it{\epsilon }}\left( k \right) = \sqrt {E(k)\left( {E(k) + 2gn} \right)} ,$$

and ${{E}}(k)=\hbar^{2}k^{2}{\!}/{\!}(2m)$. Occupation of the single-particle excited states is expressed as $\langle \hat a_k^\dagger \hat a_k \rangle = (\left| {u_k} \right|^2 + \left| {v_{ - k}} \right|^2)\, \langle\hat b_k^\dagger \hat b_k \rangle + \left| {v_{ - k}} \right|^2$. Here, the first term, proportional to $\langle \hat b_k^\dagger \hat b_k \rangle = 1/[\exp \left( {{\it{\epsilon }}\left( k \right)/k_bT} \right) - 1]$, describes the thermal depletion of the condensate. At zero temperature, *T* = 0, there is no thermal occupation of Bogoliubov quasiparticles $\langle \hat b_k^\dagger \hat b_k\rangle = 0$, and the non-zero occupation of single-particle states is due to quantum depletion $\langle \hat a_k^\dagger \hat a_k \rangle = \left| {v_{ - k}} \right|^2$. The GB states are populated due to quantum depletion, therefore the GB occupation can be expressed as *N*_GB_(*k*) = |*v*_−*k*_|^2^.

Taking the asymptotic behaviour of *N*_*GB*_, one finds that in the low wavenumber regime, which was inaccessible in our experiment, *N*_GB_$\left( k \right)\mathop {\longrightarrow }\limits^{k\xi \ll 1} \frac{{\sqrt {mgn} }}{{2\hbar k}} \propto k^{ - 1}$, and in the opposite regime $N_{\text{GB}}\left( k \right)\mathop{ \longrightarrow }\limits^{k \xi \gg 1} \frac{m^2 g^2 n^2}{{\hbar}^4 k^4} \propto k^{ - 4}$ (see details in Supplementary Note 13). The asymptotic value $\mathop {\mathrm{lim }}\nolimits_{k \to \infty } N\left( k \right)k^4 = C$ is referred to as Tan’s contact^40^, a universal quantity relating contact interactions to the thermodynamics of a many-body system. The contact in the equilibrium theory depends quadratically on peak density $C\propto n^{2},$ in agreement with the values extracted from the experiment (see Supplementary Note 11).

In the case of driven-dissipative systems, the Bogoliubov dispersion is modified and, after adiabatic elimination of the reservoir modes, the dispersion can be expressed in the analytical form:^11^

3 $${\it{\epsilon }}_{{\mathrm{neq}}}\left( k \right) = - i\frac{{\hbar {\mathrm{\Gamma }}}}{2} + \sqrt {{\it{\epsilon }}^2\left( k \right) - \left( {\frac{{\hbar {\mathrm{\Gamma }}}}{2}} \right)^2} ,$$

where the non-equilibrium relaxation parameter $\Gamma \mathop{\longrightarrow}\limits^{n\to \infty}\gamma_{\mathrm{LP}}$ and *γ*_LP_ represents the polariton decay rate. In our case, the polariton lifetime exceeds 100 ps^42^, so *ћ*Γ ≈ 3–5 μeV, which has a negligible contribution to the fitted dispersion at large wavevectors measured in this work. Therefore, for the purpose of the analysis, we use an approximation of equilibrium dispersion ${\it{\epsilon }}(k)$, Eq. (2). The fitting of the dispersions of elementary excitations is performed using a single fitting parameter, the condensate interaction energy μ = *gn*, and by taking into account the experimentally measured polariton dispersion *E*(*k*) = *E*_LP_(*k*) − *E*_LP_(0), where, $E_{{\mathrm{LP}}}(k) = \frac{1}{2}\left( {E_{\mathrm{X}} + E_{\mathrm{C}}(k) - \sqrt {\left( {\hbar {\mathrm{\Omega }}} \right)^2 + {\mathrm{\Delta }}^2(k)} } \right)$.

## Supplementary information

Supplementary Information

## Data Availability

The data that support the findings of this study are available from the corresponding author upon reasonable request.
