# Supplementary material for: Observation of quantum depletion in a non-equilibrium exciton–polariton condensate
Source: Nat Commun. 2020 Jan 22;11:429. doi: 10.1038/s41467-019-14243-6 (PMC6976592; doi:10.1038/s41467-019-14243-6)
Supplement: Supplementary file 1 — Supplementary Information [file 41467_2019_14243_MOESM1_ESM.pdf]

# Supplementary Information:

# Observation of quantum depletion in a nonequilibrium exciton-polariton condensate

**Maciej Pieczarka[1], Eliezer Estrecho[1], Maryam Boozarjmehr[1], Olivier Bleu[2], Mark Steger[3*], Kenneth West[4], Loren N. Pfeiffer[4], David W. Snoke[3], Jesper Levinsen[2], Meera M. Parish[2], Andrew G. Truscott[5], and Elena A. Ostrovskaya[1]**

[1]ARC Centre of Excellence in Future Low-Energy Electronics Technologies and Nonlinear Physics Centre, Research School of Physics, The Australian National University, Canberra, ACT 2601, Australia

[2]ARC Centre of Excellence in Future Low-Energy Electronics Technologies and School of Physics and Astronomy, Monash University, Melbourne, VIC 3800, Australia

[3]Department of Physics and Astronomy, University of Pittsburgh, Pennsylvania 15260, USA

[4]Princeton Institute for the Science and Technology of Materials (PRISM), Princeton University, Princeton, New Jersey 08544, USA

[5]Laser Physics Centre, Research School of Physics, The Australian National University, Canberra, ACT 2601, Australia

[*] Current address: National Renewable Energy Lab, Golden, Colorado 80401, USA

## Supplementary Note 1: Condensation at the excitonic detuning

Supplementary Figure 1 presents the data taken for the excitonic detuning ($\Delta = +1.8\ \mathrm{meV}$), which shows similar features to the photonic detuning case, see Figure 1 of the main text. In the power-dependent density measurements, Supplementary Figure 1a, one can observe a nonlinear growth of the density before the condensation at $k_{\parallel} = 0$ starts at around 70 mW. Similarly to the photonic detuning case, this is caused by the macroscopic occupation of excited states at lower pump power due to inefficient relaxation to the ground state. Additionally, there are macroscopically occupied high-energy modes located on top of the barrier[1], as can be seen in Supplementary Figures 1b, 1d. These modes coexist with the single-energy condensate inside the trap and manifest themselves in the peaked occupation at high $k$-vectors, see Figure 5 of the main text.

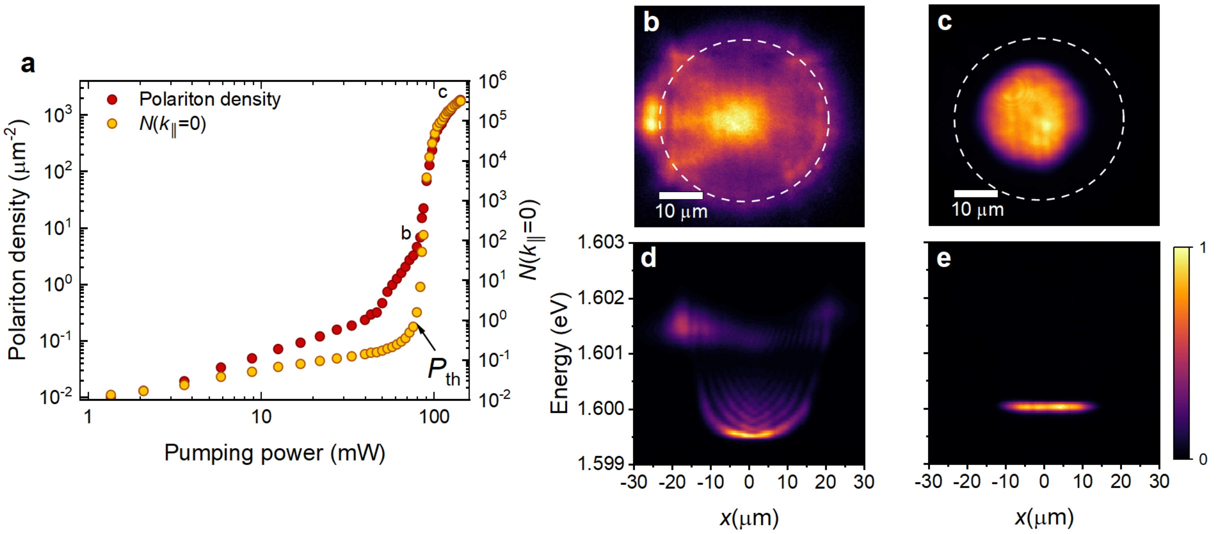


**Supplementary Figure 1 (a)** Pump power dependence of the total mean polariton density inside the trap (dark circles) and the occupation number of the $k_{\parallel} = 0$ state (light circles). Ground state condensation threshold $P_{th}$ is indicated with an arrow. **(b, c)** Energy integrated, real-space images of exciton-polariton photoluminescence at two density regimes marked in panel **(a)** shown together with **(d, e)** the corresponding real-space spectra taken in the middle of each image. **(b, d)** Intermediate density regime, $n \approx 7\ \mu\mathrm{m}^{-2}$, where the high density mode is visible together with the ground state. **(c, e)** High-density, single mode condensation, $n \approx 1848\ \mu\mathrm{m}^{-2}$.

**Supplementary Note 2: Spatial shape of the condensate at large densities**

The high-density, interaction-dominated regime is characterised by a smooth and nearly-homogeneous distribution of the ground state wavefunctions within the area defined by the optical trap. To verify this property, we extracted the real-space density distributions of condensed polaritons, which is reflected in the intensity distribution of the cavity photoluminescence taken along the real-space spectrum at the energy of the condensate, see Supplementary Figure 1e and Figure 1g in the main text. The measured profiles in the high-density regime are summarized in Supplementary Figure 2. At large densities, $n > 10^3\ \mu\mathrm{m}^{-2}$, the shape of the ground state wavefunction remains almost constant with further increase in density. The small modulations on the top of the condensate wavefunction is a result of local sample imperfections, reflecting the actual potential landscape of the sample.

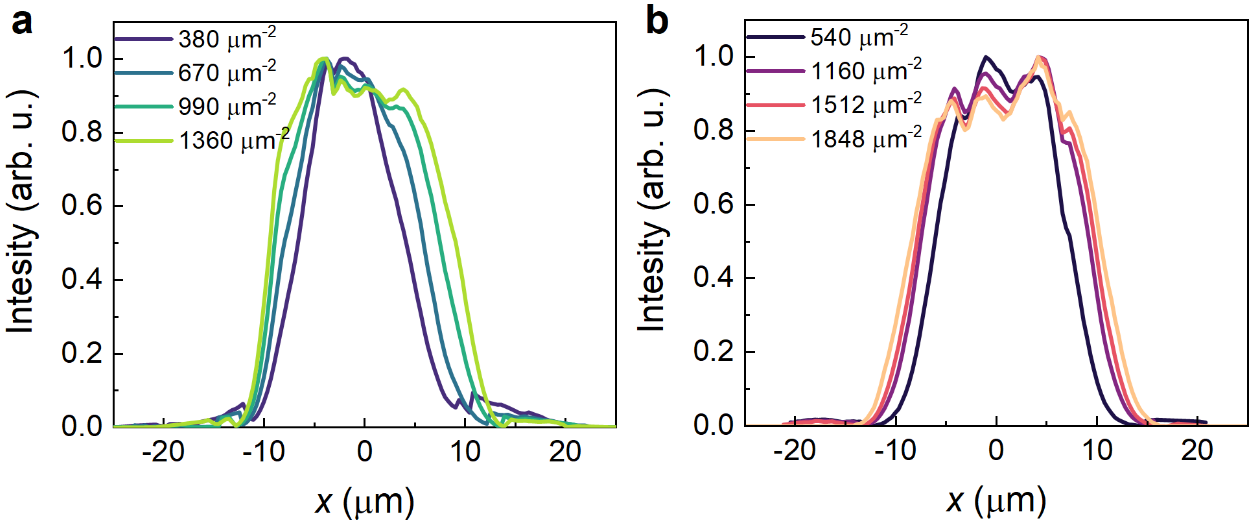


**Supplementary Figure 2** Extracted real space profiles of the condensate ground state in the high-density regime for **(a)** photonic detuning ($|X|^2 = 0.39$) and **(b)** excitonic detuning ($|X|^2 = 0.56$). Data is normalized to the maximum value.

## Supplementary Note 3: Condensate filtering in real space

To ensure that the collected photoluminescence (PL) originates from the condensate in the trap only and not from polaritons near the pump region, we use a real space filter as shown in Supplementary Figure 3a. The filter is an iris placed in the intermediate real space (near-field) image plane of the optical setup, centred with respect to the condensate position. The diameter is optimized to minimise the diffraction on its edges and to filter out the signal from the barrier region. All dispersion measurements presented in the main text were performed using this filtering technique. Supplementary Figure 3b shows the real-space spectrum when we combine the real space and the momentum space edge filters. Similarly to the momentum-resolved spectra of Figure 3 of the main text, the dominant emission originates from the condensate at $E \sim 1.600$ meV. More importantly, one can observe that the rest of the emission that constitute the NB and GB states are spread all over the trap and do not come from the barrier region. The signal outside the real space filter is due to diffraction.

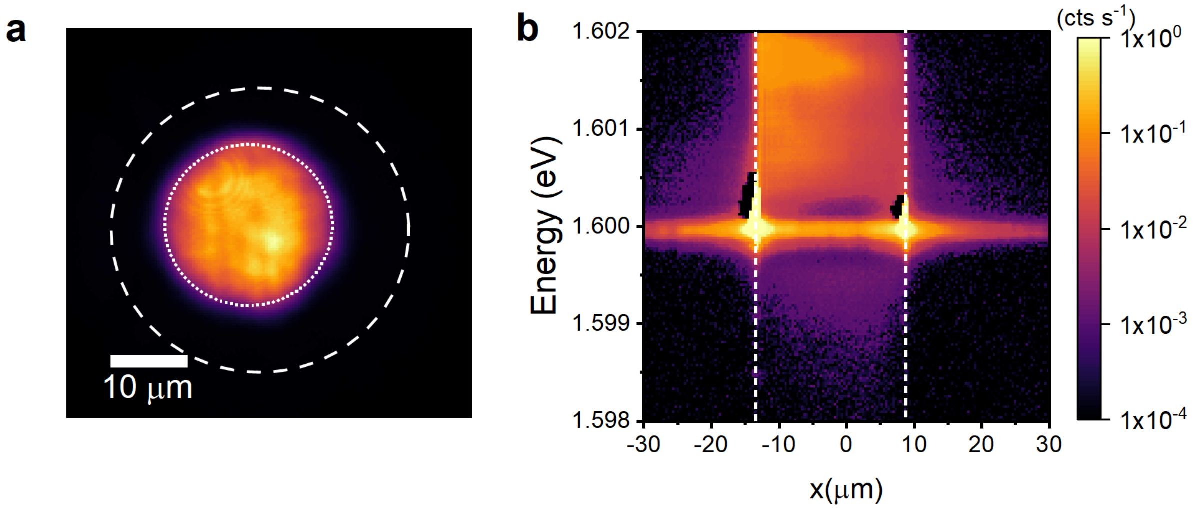


**Supplementary Figure 3 (a)** Energy integrated PL of the high-density condensate showing the edge of the real space filter (dotted circle) and the outline of the excitation profile (dashed circle). **(b)** Real space spectrum of the high-density condensate filtered in both real space (dotted lines) and momentum space (edge filter, not shown). The signal outside the real space filter is due to diffraction on the filter edges. Image is saturated and the colour scale is logarithmic.

## Supplementary Note 4: Extraction and fitting of the excitation branches

Below we provide details of the fitting procedure of the excitation spectra presented in Figure 3 of the main text. The excitation spectra in momentum space are recorded on the CCD camera, where each pixel column corresponds to a wavevector $k_{\parallel}$. Examples of spectral profile at different finite wavevectors are presented in Supplementary Figures 6a,b and 6d,e. The signal is dominated by the diffracted light from the condensate, which arises from the real space filtering. On the high-energy side, one can observe the photoluminescence of the normal branch (NB), whose spectral lineshape is irregular and is composed of the occupation of many confined excited states of the optically-induced trap. The lineshape of the NB is fit with a Voigt function to extract the intensity and energy from the peak area and centre, respectively, see Supplementary Figures 4a, 4d. The low-energy peak is the signal of the ghost branch (GB), which is much weaker, as shown in the zoomed-in plots in Supplementary Figures 4b and 4e. In contrast to the NB, the lineshapes of the GB states are smooth and are fitted to Lorentzian functions for extraction of the position and intensity of the energy peaks. The extracted energy

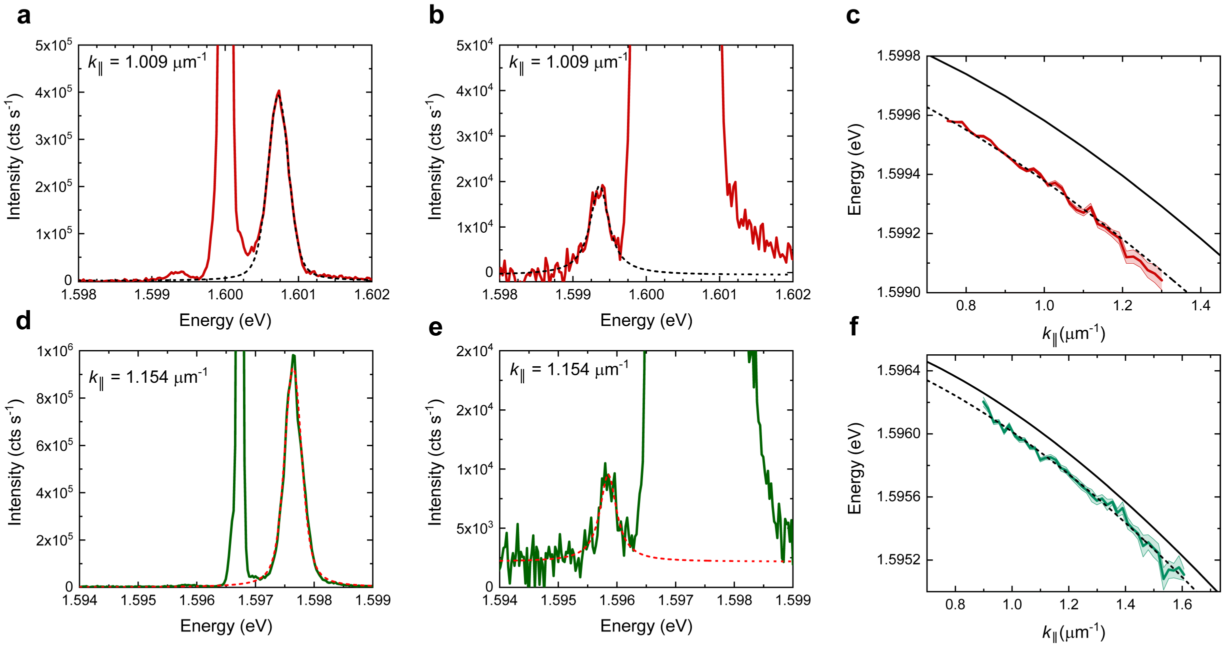


**Supplementary Figure 4** Examples of fitting to the NB and GB signals at a given wavevector for the **(a,b)** excitonic ($|X|^2 = 0.56$) and **(d,e)** photonic ($|X|^2 = 0.39$) detuning. **(a,d)** Show the full spectra, where the NB is fitted with the Voigt function (dashed line). **(b,e)** Zoomed spectra with examples of fitting to the weak GB signal with a Lorentzian lines (dashed lines). **(c,f)** Examples of fitting to the Bogoliubov spectrum of the GB (solid lines correspond to the reversed bare polariton dispersions).

dispersion of the GB is then fitted with the Bogoliubov spectrum $\epsilon(k)$ (see main text, Methods), with the condensate interaction energy $\mu = gn$ is the only fitting parameter. Examples of the renormalized spectra fits are presented in Supplementary Figures 4c and 4f. Note that we only fit the GB dispersion due to the clarity of this data, as the GB states are populated only via quantum depletion. This is unlike the NB that has additional contributions from high-energy states coming from the top of the barrier, which results in an extracted dispersion that deviates from the Bogoliubov prediction, see Figure 3 in the main text.

## Supplementary Note 5. Density-dependent blueshift of the ground state and extraction of the reservoir density

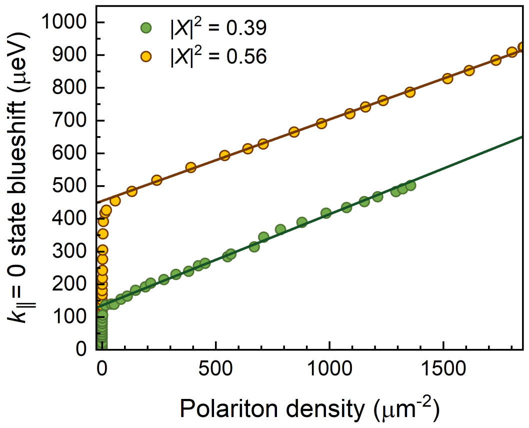


**Supplementary Figure 5** Density-dependent blueshift of the ground state extracted from the $k_\parallel = 0$ spectrum. Solid lines are linear fits to the high-density data.

Supplementary Figure 5 presents the exciton-polariton energy blueshifts extracted from the density-dependent measurements. Below the condensation threshold, at low densities, there is an anomalously large blueshift due to interaction of polaritons with the optically-injected reservoir[2,3]. At large densities above the condensation threshold, the blueshift is a linear function of the polariton density in the trap, indicating that the condensate is in the interaction-dominated Thomas-Fermi regime. We note, however, that under CW excitation the spatial depletion of the reservoir[4,8] is incomplete, which means that there is a non-negligible contribution of polariton-reservoir interactions to the blueshift within the trap. This is

manifested in larger-than-expected slopes and non-zero low-density limit, $\Delta E$, of the linear dependencies of the blueshift on density, $E = sn + \Delta E$. In the cases presented here, the slopes are: $s = 0.249 \pm 0.003\ \mu\text{eV}\mu\text{m}^2$ for the excitonic detuning and $s = 0.279 \pm 0.004\ \mu\text{eV}\mu\text{m}^2$ for the photonic detuning. These values are about two times larger than those expected to arise due to polariton-polariton interactions and the slope is larger for the photonic detuning (smaller Hopfield coefficient), which contradicts the expected behaviour of the blueshift, based on the expression of the polariton-polariton interaction energy [8-10]:

$$\mu = gn = \frac{|X|^4}{2N_{\text{QW}}} g_{\text{X}} n,$$

where $|X|^2$ is the excitonic Hopfield coefficient determining the fraction of an exciton in the exciton-polariton quasiparticle, and $g_{\text{X}} = 6E_0 a_B^2$ is the exciton-exciton interaction constant[9], with $E_0$and $a_B$ denoting the exciton binding energy and Bohr radius, respectively. The factor of 2 comes from the fact that the condensate is linearly polarized (i.e., has an equal mixture of two spin components), and the interaction of excitons with the opposite spin is negligible. The total interaction constant for total polariton density distributed amongst all quantum wells should be divided by $N_{\text{QW}}$.

The correct polariton-polariton interaction constants, $g$, were extracted from fitting the ghost branch of the Bogoliubov dispersion at various detunings and densities, using the interaction

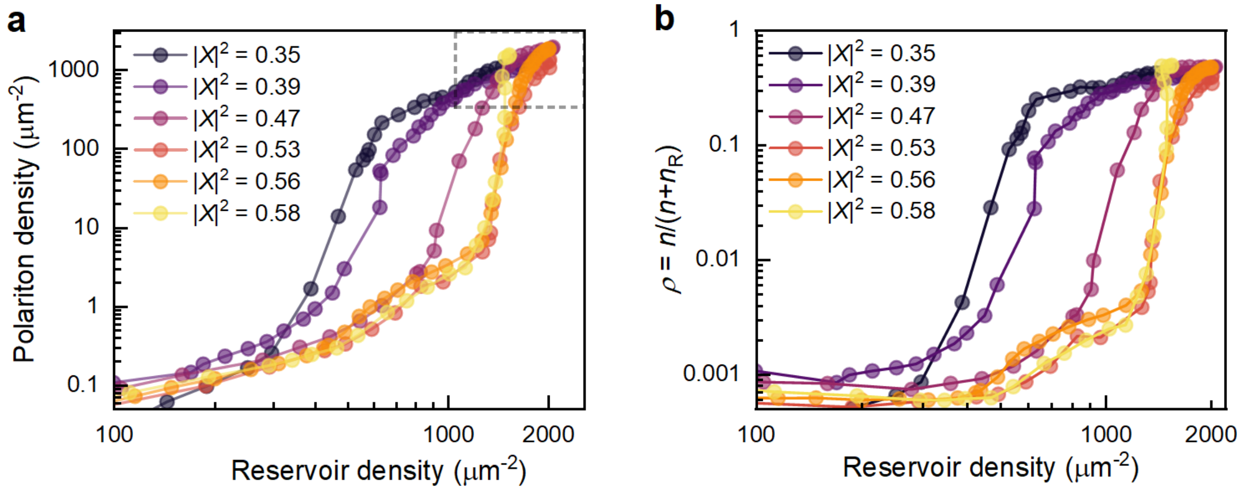


**Supplementary Figure 6 (a)** Polariton density as a function of reservoir density at different detunings (exciton fractions), extracted from a blueshift of the condensate emission energy and taking the measured polariton-polariton interaction strengths at a given detuning. The dashed rectangle indicates the density range where the GB signal is strong enough to be detected. **(b)** Condensate fraction $\rho$, calculated from the data presented in **(a).**

energy, $\mu = gn$, as a single fitting parameter (see Methods in the main text and Supplementary Note 4). This allows us to separate the contribution of the polariton-reservoir interaction to the total blueshift shown in Supplementary Figure 5. Assuming that the total blueshift of the measured $k_{\parallel} = 0$ state originates from the polariton-polariton interactions and polariton-reservoir interactions: $E = gn + g_{\mathrm{R}} n_{\mathrm{R}}$, where $g_{\mathrm{R}} = g/|X|^2$, one can extract the reservoir density, $n_{\mathrm{R}}$, at a given pumping power. Here we neglect the quantum confinement effect, which would add a correction at photonic detunings and very low polariton densities below the condensation threshold. Above the condensation threshold, where the single-mode regime is achieved, this effect has negligible impact on the blueshift[3]. The measured polariton densities as a function of extracted reservoir densities are presented in Supplementary Figure 6a. One can observe increasing reservoir densities at the polariton condensation thresholds with respect to the excitonic fraction of polaritons, reaching about 1500 $\mu\mathrm{m}^{-2}$ threshold value at excitonic detunings $|X|^2 > 0.5$. Thus, the reservoir density is non- negligible in our experiment, and the condensate fraction, defined as $\rho = n/(n + n_{\mathrm{R}})$, reaches the maximum values of around $\rho \approx 0.5$ for all detunings, see Supplementary Figure 6b. Interestingly, similar values were recently deduced by tracing the polariton condensate excitations under resonant excitation[5]. It is also important to note that the reservoir densities inside the circular trap are much smaller in comparison to excitation with a large Gaussian spot[6]. Additionally, one can point out a significant difference between the photonic and excitonic polaritons in the polariton density curves presented in Supplementary Figures 6a and 6b. At polariton densities of around $10^3\ \mu\mathrm{m}^{-2}$, where the GB signal was detectable, one reaches the gain saturation of stimulated scattering from reservoir at photonic detunings, where the growth of polariton density as a function of reservoir density is suppressed. This regime is not reached at excitonic detunings, even at the largest experimentally achievable polariton densities inside the trap.

The two types of behaviour displayed at photonic and excitonic dedunings can be qualitatively understood by employing a simplified rate-equation model for the polariton and reservoir density above the condensation threshold arising from the open-disspative Gross-Pitaevskii equation[10]:

$$\frac{dn}{dt} = -\gamma n + Rnn_{\mathrm{R}};\ \frac{dn_{\mathrm{R}}}{dt} = -\gamma_{\mathrm{R}} n_{\mathrm{R}} - Rnn_{\mathrm{R}} + P,$$

where $n, n_{\mathrm{R}}$ are the condensate and reservoir densities, $\gamma, \gamma_{\mathrm{R}}$ are the respective radiative decay rates, $R$ is the rate of stimulated scattering into the condensate mode, and $P$ is the rate of pump-driven reservoir injection. For very photonic detunings ($|X|^2 \ll 0.5$), no condensate forms at the pump region ($n = 0$), so that the reservoir density at the trap barrier continues to grow lineraly with the pump rate. A non-negligible fraction of this density, $F$, is found in the centre of the trap, with the steady state defined as $n_{\mathrm{R}}^0 \approx FP/\gamma_{\mathrm{R}}$. The steady-state condensate density determined from the rate equations as $n^0 \approx (1-F)\gamma_{\mathrm{R}}/(FR)$ is independent on the pump rate (and reservoir density) in this regime. This is the gain saturation regime that is approached at negative (photonic) detunings in Supplementary Figure 4. In the opposite regime of excitonic detunings ($|X|^2 \gg 0.5$), the condensate first forms at the pump region, which clamps the reservoir density inside the trap at a constant value $n_{\mathrm{R}}^0 \approx F\gamma/R$. However, the steady state condensate density continues to grow with the pump rate as $n^0 \approx P/F\gamma - \gamma_{\mathrm{R}}/R$. As seen in Supplementary Figure 6, this behaviour manifets itself in a steepening dependence $n^0(n_{\mathrm{R}}^0)$ at excitonic detunings.

**Supplementary Note 6: Healing length and the crossover wavevector**

The healing length of the condensate is calculated using the textbook definition $\xi = \frac{\hbar}{\sqrt{2mgn}}$, where $m$ is the polariton effective mass and $gn$ is the interaction energy extracted from the fitting of the GB excitation spectra. The calculated crossover wavevector $k_\xi = \xi^{-1}$, where the dispersion changes from linear to quadratic, is summarised in Supplementary Figure 7 for two of the detunings values probed in the experiment. The results show that our momentum-space filtering technique allows for probing the long wavevector ($k > k_\xi$) part of the elementary excitations spectrum.

.

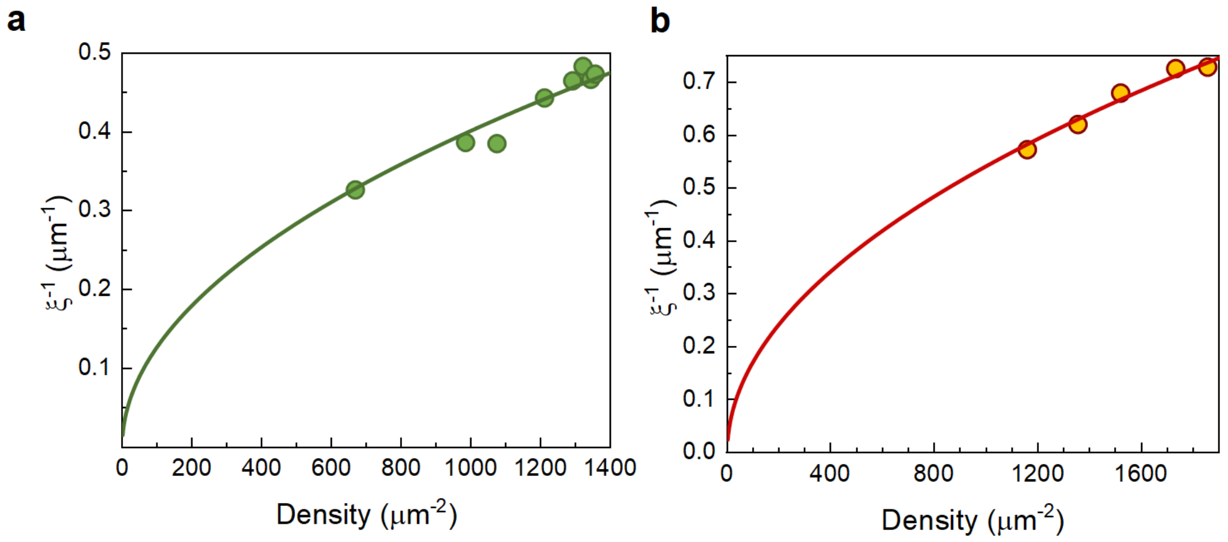


**Supplementary Figure 7** Inverse healing length of the condensate extracted for **(a)** photonic detuning ($|X|^2 = 0.39$) and **(b)** excitonic detuning ($|X|^2 = 0.56$). Solid lines are fits with a square root function.

## Supplementary Note 7: GB occupation at different positions on the sample (different detunings)

In Supplementary Figure 8, we present examples of data for different positions on the sample and different detunings showing that the observed behaviour is generic for the whole sample under similar experimental conditions. Supplementary Figure 8a illustrates data at a photonic detuning ($|X|^2 = 0.35$) where one can observe a gradual deviation from the power-law $k_{\parallel}^{-4}$ at larger densities, similar to what is presented in the main text.

In our experiment, we observe the power-law decay $k_{\parallel}^{-4}$ at all excitonic detunings, whthout any visible discrepancies within the probed wavevector range. An example at one of the detunings ($|X|^2 = 0.53$) is presented in Supplementary Figure 8b.

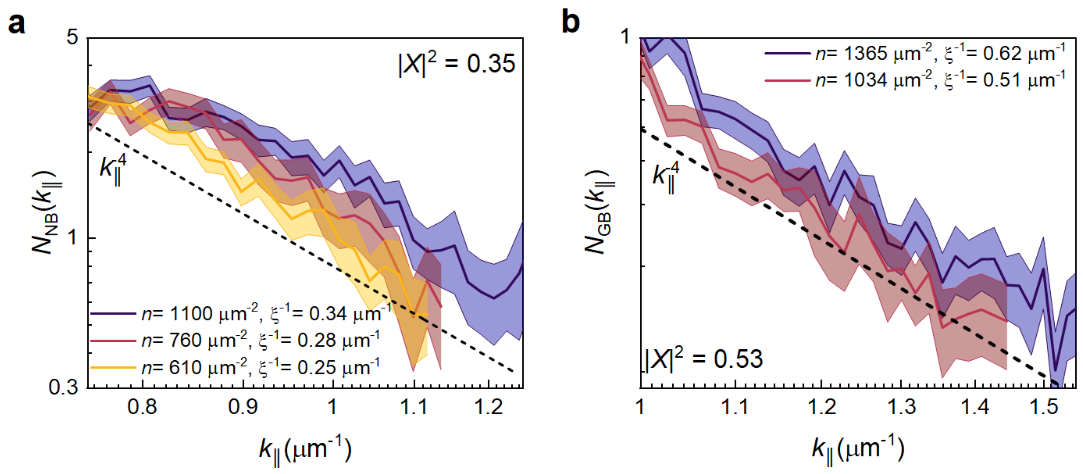


**Supplementary Figure 8** Examples of the GB occupation in momentum space at various detunings. (a) Example of a photonic detuning, where $|X|^2 = 0.35$ and densities up to $10^3\ \mu\mathrm{m}^{-2}$ were probed. (b) Example of an excitonic detuning $|X|^2 = 0.53$, where the GB occupation follows the power law $k_\parallel^{-4}$ at all probed densities. Shaded zones represent error bars of the occupation numbers extraction taking into account fitting errors of the spectra at a given $k_\parallel$.

## Supplementary Note 10. Raw data for the GB

In Supplementary Figure 9, we show raw data for the GB occupation presented in the main text (measured in photon counts per second), without recalculation to occupation per state in the momentum space (see Methods). One can observe that the power-law dependence is preserved.

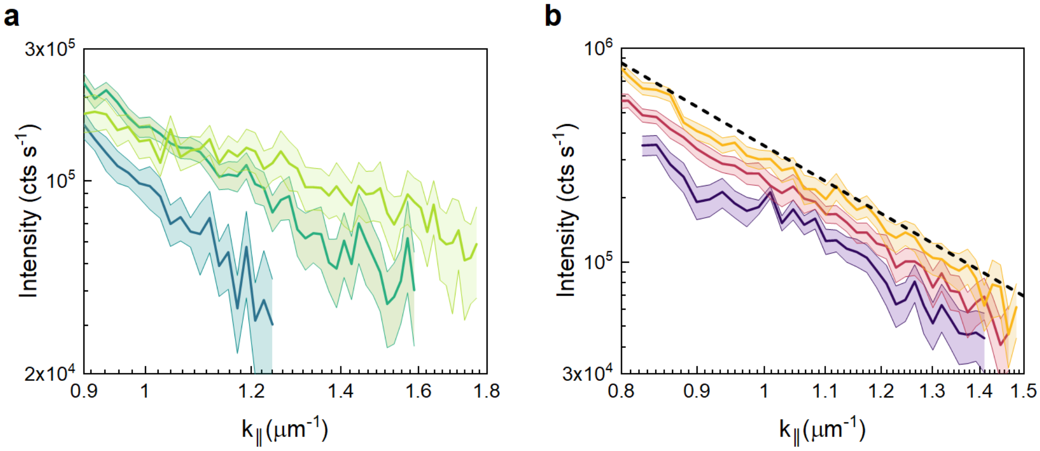


**Supplementary Figure 9** Raw data of integrated intensities for GB data presented in Fig. 5 of the main text. (a) Photonic detuning ($\Delta = -3.7\ \mathrm{meV}, |X|^2 = 0.39$) and excitonic detuning ($\Delta = +1.8\ \mathrm{meV}, |X|^2 = 0.56$). Dashed line in (b) is a guide to the eye for the power law $k_\parallel^{-4}$. One can observe that the power-law decay is also present in the raw data. Shaded zones represent error bars of the occupation numbers extraction taking into account fitting errors of the spectra at a given $k_\parallel$.

**Supplementary Note 11: Density dependence of the contact**

As described in Methods section of the main text, the direct measurement of the GB occupation in momentum space allows one to extract the value of the Tan's contact from the $N_{\mathrm{GB}}(k) \propto Ck^{-4}$ dependence at large $k$-vectors (henceforth $k$ denotes the in-plane momentum $k_{\parallel}$).

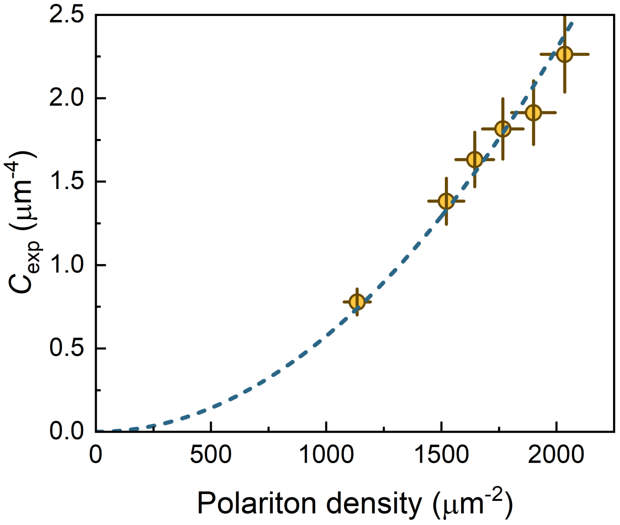


**Supplementary Figure 10** Experimentally determined values of the contact $C_{\mathrm{exp}}$ as a function of the peak polariton density determined from the real space PL spectrum. Dashed line indicates a quadratic fit to the experimental data. Error bars are determined based on the fitting to experimental data and taking into account the estimation of absolute error for the polariton density value.

Assuming the local density approximation (LDA) in the middle of the trap, where the condensate density is a smooth function, the proportionality coefficient should depend quadratically on the peak density $C \propto n^2$. Verification of this relation is presented in Supplementary Figure 10 at excitonic fraction of $|X|^2 \approx 0.56$. The peak density has been extracted from real-space spectra in the middle of the trap, to avoid averaging of the density with the periphery of the condensate. We note that the contact $\mathcal{C}_\infty$ measured for an atomic BEC, e.g., in Ref. [7], is defined as the limit of density distribution in momentum space rather than the occupation number. In a two-dimensional quantum gas, it is therefore related to the quantity defined above as $\mathcal{C}_\infty = CS/(2\pi)^2$, where $S$ is the area of the condensate in real space. Both $\mathcal{C}_\infty$ and $C$ exhibit quadratic dependence on the peak density in the LDA.

**Supplementary Note 12: Domain of dynamical instability**

In certain regions of system parameters, spatially homogeneous condensates created in the CW regime were predicted to exhibit dynamical (modulational) instability, which is driven by fluctuations of the reservoir[11,12]. This instability was experimentally observed in large, quasi-1D condensates[13]. Analytical estimate obtained using the open-dissipative Gross-Pitaevskii model[11] yields the instability domain for the homogeneous pump rate (i.e. the rate of reservoir replenishing): $P/P_{\mathrm{th}} < P_{\mathrm{MI}}/P_{\mathrm{th}} = \gamma g_{\mathrm{R}}/(g\gamma_R)$, where the threshold pump rate is defined as $P_{\mathrm{th}} = \gamma\gamma_{\mathrm{R}}/R$ (other parameters are defined in Supplementary Note 5). The boundary of the instability region can be expressed as a function of the excitonic Hopfield coefficient:

$$P_{\mathrm{MI}}/P_{\mathrm{th}} = 1 + \frac{\gamma_{\mathrm{C}}}{\gamma_{\mathrm{X}}}\frac{(1-|X|^2)}{|X|^2}, \qquad (1)$$

where we have assumed that the decay rate of highly excitonic reservoir particles is equal to that of excitons, and $\gamma_{\mathrm{C}}$ is the decay rate of cavity photons. For our long-lifetime system, the ratio $\gamma_{\mathrm{C}}/\gamma_{\mathrm{X}} \approx 10$ , and the corresponding instability domain is shown in Supplementary Figure 11a. In this domain, the elementary excitations within the band of wavevectors[11] $k/k_\xi < 2\sqrt{P_{\mathrm{MI}}/P - 1}$, shown in Supplementary Figure 11b, may grow exponentially. As pointed out in Ref[14], this means that the fluctuations become large, the adiabatic approximation for the reservoir dynamics (which is the basis for Eq. (3) in Methods) breaks down, and the population of the GB departs from the Bogoliubov prediction. Numerical simulations of the open-dissipative mean field model show deviation of the $N(k)$ from the Bogoliubov prediction and characteristic flattening of the momentum dependence[14,15], however the details of this behaviour are model-specific.

The domain of modulational instability is strongly suppressed for spatially inhomogeneous condensates[16]. However, our trapped condensates created at photonic detunings ($|X|^2 < 0.5$) approach the “flat-top”, spatially homogeneous profile shapes at large pump powers and large densities (see, e.g., Supplementary Figure 2a). It is therefore possible that, as we increase the pump power, we drive the system into the modulational instability domain[14], where large deviations from the Bogoliubov distribution $N(k)$ are expected. Indeed, such deviations are

observed in our experiment (see Fig. 5 of the main text and Supplementary Figure 8a). Nevertheless, we do not observe strong density modulations of the condensate typically associated with the dynamical instability[13], which requires further investigation of the relevance of this regime and the corresponding mean-field models to our observations.

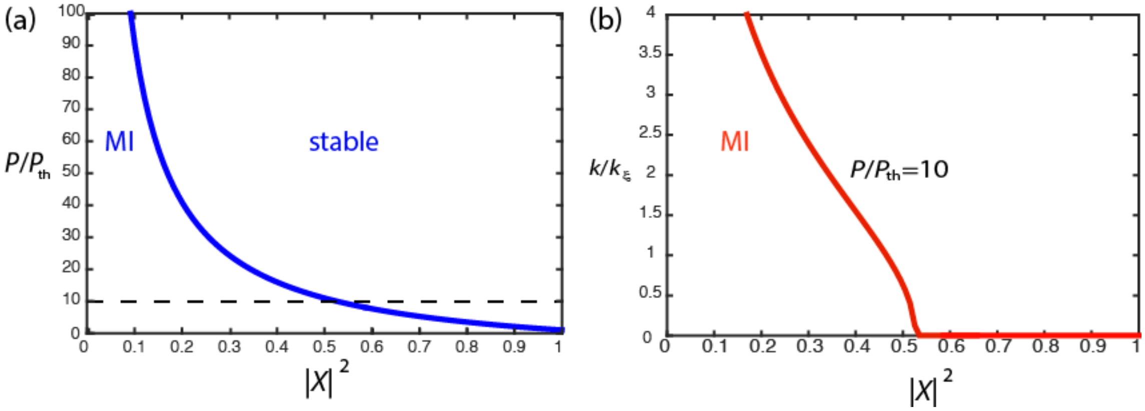


**Supplementary Figure 11** (a) Boundary of the dynamical **(**modulational) instability of a homogeneous CW condensate given by Eq. (1). (b) Domain of unstable excitation wavevectors corresponding to the pump rate relative to the condensation threshold indicated in (a) by a horizontal dashed line.

**Supplementary Note 13: Polaritons vs massive bosons: influence of non-parabolicity of the polariton dispersion**

When probing the elementary excitations of the condensate in a large momentum limit, it is important to understand to what extent the non-parabolicity of the lower polariton dispersion, $E_{\mathrm{LP}}(k)$, which may become prominent in this limit, modifies the expressions for the energy of the Bogoliubov excitations used in the main text. We recall that the energy of the lower polariton branch, derived from the standard coupled exciton-photon model[10], takes the form:

$$E_{LP}(k) = \frac{1}{2}\left[E_{\mathrm{C}}(k) + E_{\mathrm{X}} - \sqrt{\Delta^2(k) + E_R^2}\right], \quad (2)$$

where $E_{\mathrm{C}}(k) = E_{\mathrm{C}}(0) + \hbar^2 k^2/2m_{\mathrm{C}}$ is the cavity photon dispersion, $m_{\mathrm{C}}$ is the effective mass of the cavity photon, $E_{\mathrm{X}}$ is the exciton energy (assumed constant), $\Delta(k) = E_{\mathrm{C}}(k) - E_{\mathrm{X}}$ is the exciton-photon detuning ranging from positive (excitonic) to negative (photonic), and $E_{\mathrm{R}} = 2\hbar\Omega$ is the Rabi splitting at zero detuning.

Taking into account the polariton energy in momentum space, $E_{\mathrm{LP}}(k)$, the energy of the elementary excitations is expressed as follows:

$$\epsilon^{\mathrm{LP}}(k)=\sqrt{T_{\mathrm{LP}}(k)}\sqrt{T_{\mathrm{LP}}(k)+2\mu}\,,\quad (3)$$

where $T_{\mathrm{LP}}(k)=E_{\mathrm{LP}}(k)-E_{\mathrm{LP}}(0)$ , and $\mu=gn$. We note that the standard expression for the Bogoliubov dispersion in equilibrium (Eq. (2) in the main text, Methods) is recovered from Eq. (3) by replacing the non-parabolic kinetic energy of the lower polariton $T_{\mathrm{LP}}(k)$ with the parabolic (effective mass) approximation $T_{\mathrm{LP}}(k)\to E(k)=\hbar^2k^2/2m$, where $m$ is the effective mass of the lower polariton, as defined in the main text. The crossover wavevector, which is defined by the healing length as the value of $k_\xi=\xi^{-1}$, at which the transition from the phonon to the free-particle behaviour of elementary excitations occurs, i.e. $T_{\mathrm{LP}}(k_\xi)=\mu$. Just as in the case of massive bosons, the large wavevector limit is then defined as $k\gg k_\xi$, or equivalently $T_{\mathrm{LP}}(k)\gg\mu$.

The expression for the amplitude of the elementary excitations can then be written as

$$u_k^{\mathrm{LP}},v_{-k}^{\mathrm{LP}}=\pm\frac{1}{\sqrt{2\epsilon^{\mathrm{LP}}(k)}}\sqrt{T_{\mathrm{LP}}(k)+\mu\pm\epsilon^{\mathrm{LP}}(k)}\,,\quad (4)$$

and the occupation of the ghost branch is:

$$N_{\mathrm{GB}}^{\mathrm{LP}}(k)=\left|v_{-k}^{\mathrm{LP}}\right|^2=\frac{1}{2}\frac{1+\mu/T_{\mathrm{LP}}(k)}{\sqrt{1+2\,\mu/T_{\mathrm{LP}}(k)}}-\frac{1}{2}\,,\quad (5)$$

where the ratio $\mu/T_{\mathrm{LP}}(k)$ defines the ratio of the wavevectors $k_\xi^2/k^2=\mu/T_{\mathrm{LP}}(k)$ for the case of non-parabolic LP dispersion. In the limit of large momenta, $T_{\mathrm{LP}}(k)\gg\mu$, one can expand Eq. (5) in the powers of the small parameter $\mu/T_{\mathrm{LP}}(k)\ll 1$. Omitting the higher-order terms allows us to obtain the asymptotic behaviour at large momenta:

$$N_{\mathrm{GB}}^{\mathrm{LP}}(k)\xrightarrow{k\xi\gg 1}\frac{\mu^2}{4T_{\mathrm{LP}}^2(k)}=\frac{g^2n^2}{4[E_{\mathrm{LP}}(k)-E_{\mathrm{LP}}(0)]^2}.\quad (6)$$

This function tends to a non-zero, constant value at very large momenta:

$$N_{\mathrm{GB}}^{\mathrm{LP}}(k)\xrightarrow{k\to\infty}\frac{g^2n^2}{4[E_{\mathrm{X}}-E_{\mathrm{LP}}(0)]^2}=\frac{g^2n^2}{\left[\sqrt{\Delta^2(0)+E_{\mathrm{R}}^2}-\Delta(0)\right]^2}\,,\quad (7)$$

which differs from the corresponding behaviour for a massive boson:

$$N_{\mathrm{GB}}(k) \xrightarrow{k\xi \gg 1} \frac{\mu^2}{4T^2(k)} = \frac{g^2 n^2}{4|C|^4[E_{\mathrm{C}}(k) - E_{\mathrm{C}}(0)]^2} = \frac{m^2 g^2 n^2}{\hbar^4 k^4} \propto k^{-4}\,, \quad (8)$$

where $|C|^2 = 1 - |X|^2$ is the photonic Hopfield coefficient (photon fraction) at $k = 0$. However, for the range of the wavevectors and detunings probed in the experiment, the discrepancy between the asymptotic behaviour of $N_{\mathrm{GB}}(k)$ and $N_{\mathrm{GB}}^{\mathrm{LP}}(k)$ given by Eqs. (6) and (8), respectively, is small. The long-scale and the experimentally relevant shorter scale ($k^2/k_\xi^2 > 1$) behaviour of the two functions is illustrated in the Supplementary Figures 12-14, with $k_\xi$ defined by the condition $T_{\mathrm{LP}}(k_\xi) = \mu$.

We note that the departure of $N_{\mathrm{GB}}^{\mathrm{LP}}(k)$ from $\propto k^{-4}$ asymptote at large momenta due to the non-parabolicity of the polariton dispersion cannot explain the observed behaviour for photonic detunings, since $N_{\mathrm{GB}}^{\mathrm{LP}}(k) \longrightarrow N_{\mathrm{GB}}(k)$ for $k^2/k_\xi^2 > 1$ as $|C|^2 \longrightarrow 1$. As shown in Supplementary Figure 13, the fit to the $\propto k^{-4}$ asymptote should improve for photonic detunings, which is contrary to what is observed in the experiment (cf. Figure 5e in the main text, the highest density data shown in light green). As noted in Methods of the main text, this effect also does not affect the fitting of the experimentally measured ghost branch presented in Supplementary Note 4, since the actual, experimentally measured lower polariton dispersion $E_{\mathrm{LP}}(k)$ was used in the fitting, rather than its parabolic (effective mass) approximation.

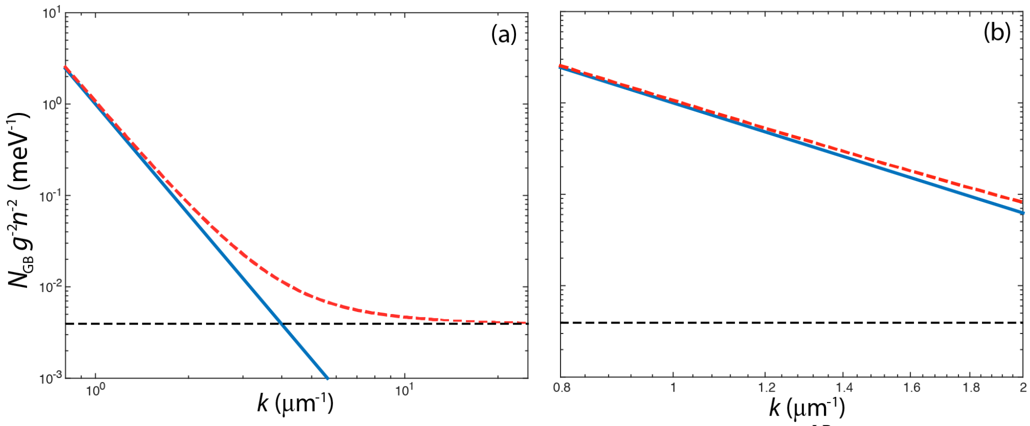


**Supplementary Figure 12** Comparison between the asymptotic behavior of $N_{GB}^{LP}(k)$ given by Eq. (6) (red dashed), and the asymptotic behavior of $N_{GB}(k) \propto k^{-4}$ given by Eq. (8) (blue) at (a) very long, $k^2/k_\xi^2 \gg 1$, and (b) shorter, $k^2/k_\xi^2 > 1$, ranges of the wavevector for $\Delta = 0\ meV$, $|X|^2 = 0.5$. The horizontal black dashed line is given by Eq. (7).

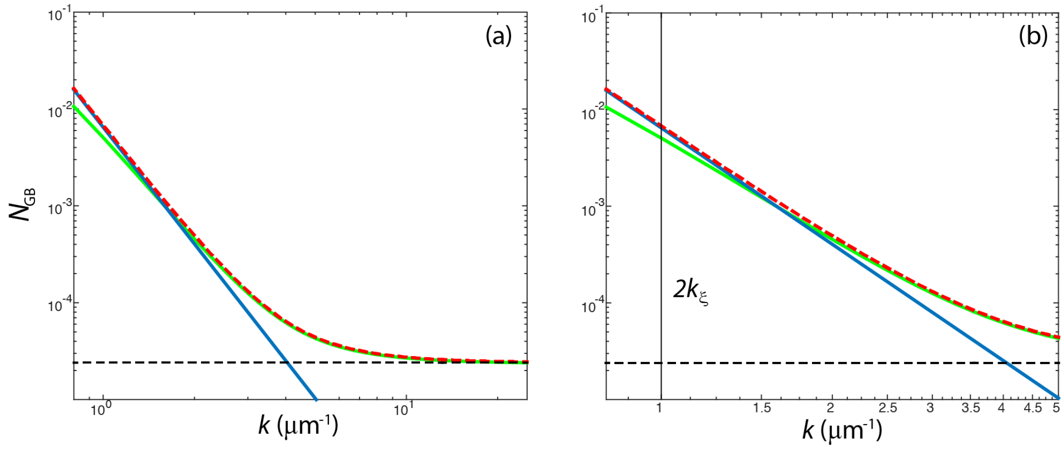


**Supplementary Figure 13** Comparison of the behaviour of $N_{GB}^{LP}(k)$ given by Eq. (5) (light green), its asymptotic behaviour given by Eq. (6) (red dashed), and the asymptotic behaviour of $N_{GB}(k) \propto k^{-4}$ given by Eq. (8) (blue) at (a) very long, $k^2/k_\xi^2 \gg 1$, and (b) shorter, $k^2/k_\xi^2 > 1$, ranges of the wavevector for $\Delta = -3.7\ meV$, $|X|^2 = 0.39, k_\xi = 0.51\ \mu m^{-1}$. The horizontal black dashed line is given by Eq. (7).

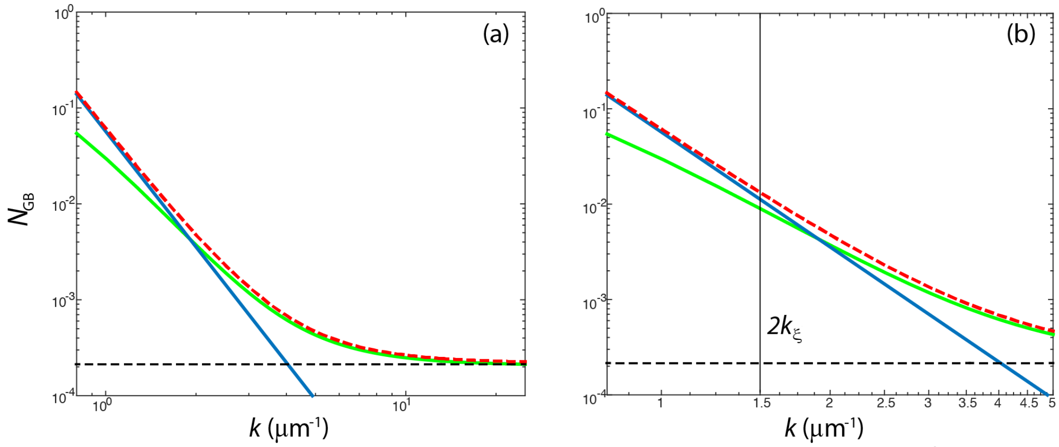


**Supplementary Figure 14** Same as Supplementary Figure 13, but for $\Delta = +1.8$ meV, $|X|^2 = 0.56, \mathrm{k}_\xi = 0.76\ \mu\mathrm{m}^{-1}$.

## Supplementary References


1. Ballarini, D. *et al.* Macroscopic Two-Dimensional Polariton Condensates. *Phys. Rev. Lett.* **118**, 215301 (2017).
2. Sun, Y. *et al.* Direct measurement of polariton-polariton interaction strength. *Nat. Phys.* **13**, 870–875 (2017).
3. Pieczarka, M. *et al.* Effect of optically-induced potential on the energy of trapped exciton-polaritons below the condensation threshold. *Phys. Rev. B* **100**, 085301 (2019).
4. Estrecho, E. *et al.* Single-shot condensation of exciton polaritons and the hole burning effect. *Nat. Commun.* **9**, 2944 (2018).
5. Stepanov, P. *et al.* Dispersion relation of the collective excitations in a resonantly driven polariton fluid. *Nat. Commun.* **10**, 3869 (2019).
6. Deng, H.*et al*. Polariton lasing vs. photon lasing in a semiconductor microcavity. *Proc. Natl. Acad. Sci.* **100**, 15318–15323 (2003).
7. Chang, R. *et al.* Momentum-Resolved Observation of Thermal and Quantum Depletion in a Bose Gas. *Phys. Rev. Lett.* **117**, 235303 (2016).
8. Estrecho, E. Direct measurement of polariton-polariton interaction strength in the Thomas-Fermi regime of exciton-polariton condensation. *et al. Phys. Rev. B* **100**, 035306 (2019).
9. Tassone, F. & Yamamoto, Y. Exciton-exciton scattering dynamics in a semiconductor microcavity and stimulated scattering into polaritons, *Phys. Rev. B* **59**, 10830 (1999).
10. Carusotto, I. & Ciuti, C. Quantum fluids of light. *Rev. Mod. Phys.* **85**, 299–366 (2013).
11. Smirnov, L. A. *et al*. Dynamics and stability of dark solitons in exciton-polariton condensates, Phys. Rev. B **89**, 235310 (2014).
12. Liew, T. C. H. *et al*. Instability-induced formation and nonequilibrium dynamics of phase defects in polariton condensates, Phys. Rev. B **91**, 085413 (2015).
13. Baboux, F. *et al*., Unstable and stable regimes of polariton condensation, Optica **5**, 1163 (2018).
14. Bobrovska, N. & Matuszewski, M. Adiabatic approximation and fluctuations in exciton-polariton condensates, Phys. Rev. B **92**, 035311 (2015).
15. Chiocchetta, A. & Carusotto, I. Quantum Langevin model for nonequilibrium condensation. *Phys. Rev. A* **90**, 23633 (2014).
16. Bobrovska, N. *et al*., Stability and spatial coherence of nonresonantly pumped exciton-polariton condensates, Phys. Rev. B **90**, 205304 (2014).
